# A Joint Symbol-Detection, Channel-Estimation and Decoding Scheme under Few-Bit ADCs in mmWave Communications

**DOI:** 10.3390/s20071857

**Published:** 2020-03-27

**Authors:** Peng Sun, Fei Liu, Jianhua Cui, Wei Wang, Yangdong Ye, Zhongyong Wang

**Affiliations:** 1 School of Information Engineering, Zhengzhou University, Zhengzhou 450000, China; iepengsun@zzu.edu.cn (P.S.); ieliufei@gs.zzu.edu.cn (F.L.); iewwang@zzu.edu.cn (W.W.); ieydye@zzu.edu.cn (Y.Y.); 2 School of Physics and Electronic Information, Luoyang Normal University, Luoyang 471934, China; jhcuizzu@foxmail.com

**Keywords:** IoT, mmWave communications, few-bit ADC, parametric bilinear generalized approximate message passing, doping factor

## Abstract

Few-bit analog-to-digital converter (ADC) is regarded as a promising technique to greatly reduce power consumption of Internet of Things (IoT) devices in millimeter-wave (mmWave) communications. In this work, based on the recently proposed parametric bilinear generalized approximate message passing (PBiGAMP), we propose a new scheme to perform joint symbol detection, channel estimation and decoding. The proposed scheme is flexible to deal with discrete prior on symbols, Gaussian mixture prior on channels and quantized likelihood on observations. Furthermore, we introduce doping factor to control the portion of “extrinsic” and “posterior” information with negligible complexity increase. Since this joint scheme can be implemented via fast Fourier transformation (FFT), the complexity grows only logarithmically. Compared to the benchmark algorithms, numerical results show that the proposed joint scheme can achieve significant performance gain, and demonstrate the effectiveness in dealing with the nonlinear distortion from few-bit ADC.

## 1. Introduction

Compared to current 4G Long Term Evolution (LTE) networks, the vision of next generation 5G wireless communications lies in providing very high data rates, extremely low latency, and manifold increase in base station capacity [1]. In particular, 5G wireless networks are likely to incorporate Millimeter-wave (mmWave) technology [2], which exploits large chunks of bandwidth at carrier frequencies of 30 GHz and above [3]. In addition, the development of 5G networks is driven by future Internet of Things (IoT) connectivity [4].

However, the main challenge in mmWave systems comes from the analog-to-digital converters (ADCs) used at IoT receivers, whose power consumption grows exponentially with the number of bits used in conversion. Specially, at GHz bandwidths, high-precision (e.g., 10 bit) ADCs may consume several watts of power, which is unrealistic for handheld mobile IoT devices. Another challenge is that high-precision ADCs may be too costly in hardware implementation. Therefore, there has been a growing interest in few-bit (e.g., 1–3 bit) ADCs at receiver side. However, few-bit ADCs will introduce severe quantization distortion to receiving signals, which brings difficulties in receiver design.

Furthermore, wide bandwidth will also lead to challenges in transmitters. In particular, the wide-bandwidth linear amplifiers are too costly and power-hungry, which suggests to transmit signals with low peak-to-average power (PAPR) ratio. Compared to orthogonal frequency division multiplexing (OFDM) [5], single-carrier (SC) with frequency domain equalization (FDE) [6,7] has similar performance and complexity but much lower PAPR, which relaxes requirements on power-amplifier linearity and thus enables the use of more efficient and cheaper amplifiers. Due to the above reasons, we consider SC system with few-bit ADCs in this work.

We now review relevant existing work on receiver design under few-bit quantization. Usually, channel estimation [8,9,10] and symbol detection [11,12,13] are separately considered. Particularly, in Reference [8], a broadband channel estimation algorithm was proposed in a multiple input multiple output (MIMO) system based on generalized approximate message passing (GAMP) [14] and vector approximate message passing (VAMP) [15]. However, this paper only focused on channel estimation without considering symbol detection. In Reference [11], the authors proposed a computationally efficient method using GAMP and fast Fourier transform (FFT) in large-scale MIMO uplink system, where perfect channel state information (PCSI) was assumed to be known at receiver. In recent years, researchers show that joint channel estimation and symbol detection, even involving bit decoding, can significantly improve performance. In Reference [16], a Bayes-optimal joint channel-and-data estimation was proposed by employing bilinear GAMP (BiGAMP) [17]. In Reference [18], a joint channel-and-data estimation was realized via a Turbo-like approach, where GAMP was used twice in channel estimation and data detection, respectively. However, both Reference [16] and Reference [18] only consider flat fading channels, and wideband channels are frequency selective in practice. In Reference [19], a joint channel-estimation, symbol-detection and decoding scheme is proposed using approximate message passing. Nevertheless, Reference [19] requires OFDM which has high PAPR. Considering the quantized SC systems with frequency selective channels, our recent work in Reference [20] designed a joint receiving scheme based on the parametric bilinear generalized approximate message passing (PBiGMAP) [21].

In this work, we further apply PBiGAMP into the receiver design in the mmWave communication systems under few-bit ADCs. We propose a joint symbol detection, channel estimation and decoding scheme, which can be implemented in a fast way via FFTs. This scheme is compatible with the Gaussian mixture model to estimate sparse mmWave channels, the discrete prior to detect transmitting symbols and non-linear likelihood to cope with quantization distortion from few-bit ADCs. The main contributions of this work are in the followings:

- We regard PBiGAMP’s quantities as noise corrupted versions of true parameters to be estimated, helping understand the inner behavior of PBiGAMP.
- Different from the common sense about Turbo equalization [22], the proposed scheme introduces doping factor to control the portion of “extrinsic” and “posterior” information with negligible complexity increase, which can include the joint approach in Reference [20] as a special case.
- Numerical results show that the proposed scheme can obtain significant performance gain compared to the benchmark algorithms.

The rest of this paper is organized as follows: In Section 2, we present the SC Model under few-bit ADCs and the corresponding factor graph representation. Section 3 describes the proposed joint symbol-detection, channel-estimation and decoding scheme. Section 4 outlines the simulation results. Finally, Section 5 concludes this paper.

***Notation:*** We use boldface uppercase letters like B to denote matrices and boldface lowercase letters like b to denote vectors, where $b_{i}$ represents the *i*th element of b, and ${\left[B\right]}_{i,j}$ represents the *i*th row and *j*th column of B. Also, $I_{M}$ is the M×M identity matrix, $1_{M}$ is the *M*-length vector of ones, $0_{M}$ is the *M*-length vector of zeros, Diag(b) is the diagonal matrix formed from the vector b, diag(B) is the vector formed from the diagonal of matrix B, $F_{N}$ is the N×N unitary discrete Fourier transform (DFT) matrix, $F_{N}^{1:L}$ is the matrix formed by the first *L* columns of $F_{N}$. For matrices and vectors, ${\left(\cdot \right)}^{T}$ denotes transpose, ${\left(\cdot \right)}^{H}$ denotes conjugate transpose, ${\left(\cdot \right)}^{*}$ denotes conjugate, and ⊗ denotes the Kronecker product. Likewise, ⊙ denote element-wise multiplication. Finally, the probability density function (pdf) of a multivariate complex Gaussian random vector x with mean $\hat{x}$ and covariance Σ will be denoted by $\mathrm{CN}(x;\hat{x},\Sigma )$.

## 2. System Model and Factor Graph Representation

### 2.1. Single-Carrier Block Transmission System

Considering single-carrier block transmission system, where $N_{b}$ information bit $b\in {\left\{0,1\right\}}^{N_{b}}$ is encoded and interleaved to a coded sequence $c\in {\left\{0,1\right\}}^{N_{c}}$ of length $N_{c}$, which is then mapped to data symbol $x_{D}\in S^{N_{D}}$ with S being a $2^{A}$-ary complex symbol alphabet. Data symbol $x_{D}$, pilot symbol and guard symbol are further collected into the transmitted matrix $X\in C^{M\times K}$, where the first $K_{P}$ columns contain pilot samples and the remaining $K-K_{P}$ columns contain “data+guard” samples.

The unquantized received samples $\bar{Y}$ can be represented as

(1) $$\begin{array}{cc} \bar{Y} & =HX+W\in C^{M\times K}, \end{array}$$

where $H\in C^{M\times M}$ is the circulant matrix with first column ${\left[h^{T}\;0_{M-L}^{T}\right]}^{T}$, and $h≜{\left[h_{0},\ldots ,h_{L-1}\right]}^{T}$ is the baseband channel impulse response, and $W\in C^{M\times K}$ contains additive white Gaussian noise (AWGN) with variance $\sigma_{w}^{2}$, which is assumed to be known. We can then write Equation (1) in vectorized form as

(2) $$\begin{array}{c} \bar{y}=\left(I_{K}⊗H\right)x+w, \end{array}$$

with $\bar{y}≜\mathrm{vec}\left(\bar{Y}\right)$, x≜vec(X), w≜vec(W), and ⊗ denoting the Kronecker product.

**Figure 1:**
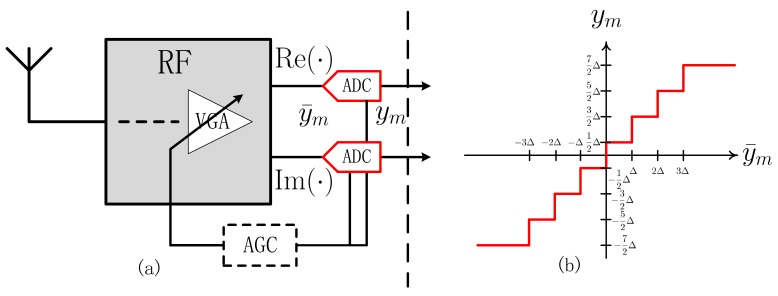
A quantized system with few-bit ADC. (**a**) Radio frequency (RF) architecture on receiver side. (**b**) An example of b=3 -bit quantization.

As illustrated in Figure 1, on the receiver side, a variable gain amplifier (VGA) with an automatic gain control (AGC) is used before quantization to ensure that analog baseband samples are within a proper range, for example, (−1,+1). In the sequel, the received signal is down-converted into analog baseband samples $\bar{y}$ and then discretized using a complex-valued quantizer Q(·), yielding the quantized received samples

(3) $$\begin{array}{c} y=Q\left(\bar{y}\right), \end{array}$$

where the few-bit quantizer Q(·) applies component-wise and we assume in our numerical experiments that *b*-bit uniform mid-rise quantization [23] is separately applied to the real and imaginary parts. In particular, the *m*-th entry in y can be represented as

(4) ym=sign(Re(y¯m))min|Re(y¯m)|△Re,2b−1−12+jsign(Im(y¯m))min|Im(y¯m)|△Im,2b−1−12,

where △Re≜ERe(y¯m)2△b, △Im≜EIm(y¯m)2△b, and ${△}_{b}$ is chosen to minimize the mean-squared error (MSE) $E\left[|y_{m}-{\bar{y}}_{m}{|}^{2}\right]$ under Gaussian ${\bar{y}}_{m}$. The average powers ERe(y¯m)2 and EIm(y¯m)2 can be measured by analog circuits before the ADC. When b>1, such measurements are typically performed as part of AGC.

### 2.2. System Factor Graph

Our goal is to infer the information bits b from the few-bit measurements y under the block-transmission model in Equation (1) and the few-bit quantization model in Equation (4). Particularly, the posterior bit marginals $p(b_{i}|y)$ can in principle be computed via

(5) $$\begin{array}{cc} p(b_{i}|y)\; & =\sum_{b_{-i}}p\left(b|y\right)=\sum_{b_{-i}}\frac{p(y|b)p(b)}{p(y)}\propto \sum_{b_{-i}}p\left(y|b\right) \end{array}$$

(6) $$\begin{array}{cc} \; & =\sum_{b_{-i},x,c}\int_{C^{L}}p\left(y|h,x\right)p\left(h\right)p\left(x|c\right)p\left(c|b\right)\;dh\\ \; & =\sum_{b_{-i},c}p\left(c|b\right)\sum_{x}\int_{C^{L}}\left[\prod_{m=1}^{MK}p\left(y_{m}|h,x\right)\right]\left[\prod_{l=0}^{L-1}p\left(h_{l}\right)\right]\;dh \end{array}$$

(7) $$\begin{array}{cc} \; & \;\times \left[\prod_{k=1}^{K_{D}}\prod_{n=0}^{N_{D}-1}p\left(x_{(K_{P}+k-1)M+n}|c_{\left(k-1\right)N_{D}+n}\right)\right], \end{array}$$

where p(y|h,x), p(h), p(x|c) and p(c|b) denote observation likelihood, channel prior, symbol mapping and coding/interleaving constraint, respectively, and $b_{-i}≜{\left[b_{1},\ldots ,b_{i-1},b_{i+1},\ldots ,b_{N_{b}}\right]}^{T}$. Above, Equation (5) can be reached due to the uniformly distributed assumption on information bits b and Bayes’ rule; Equation (6) is due to the dependency relationships among the random vectors y, h, x, c, and b; and Equation (7) is due to the separable nature of p(y|h,x), p(h), and p(x|c).

**Figure 2:**
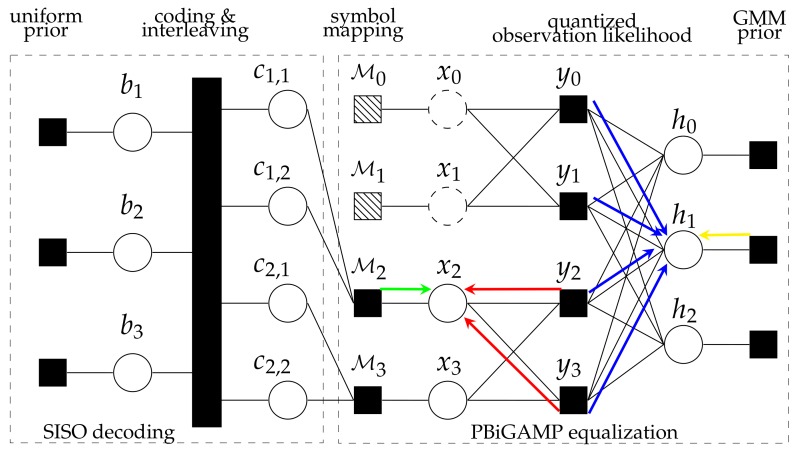
The factor graph corresponding to the single-carrier transmission.

We can obtain the exact posterior bit marginal distribution $p(b_{i}\;|\;y)$ in principle, but doing so is impractical from the standpoint of complexity. A practical alternative is to perform belief-propagation (BP) using the sum-product algorithm (SPA) [24] on the factor graph. The above communication systems can be visualized using bipartite factor graph shown in Figure 2, where the solid rectangles represent the factor nodes and the open circles represent the variable nodes. The factor graph can be partitioned into two subgraphs: the left subgraph corresponds to soft-input and soft-output (SISO) decoding and the right subgraph corresponds to soft equalization with unknown channels. However, exact implementation of the SPA in Figure 2 is still intractable in the soft-equalization subgraph. As a computationally efficient approximation of the SPA, the recently proposed PBiGAMP [21] approaches to the marginal posteriors of x and h iteratively from their noisy bilinear observation y under independent assumptions on ${\left\{p_{x_{n}}\left(x_{n}\right)\right\}}_{n=0}^{MK-1}$, ${\left\{p_{h_{l}}\left(h_{l}\right)\right\}}_{l=0}^{L-1}$ and ${\left\{p_{y_{m}/z_{m}}\left(y_{m}/z_{m}\right)\right\}}_{m=0}^{MK-1}$.

## 3. Joint Symbol Detection, Channel Estimation and Decoding Scheme

### 3.1. Review of PBiGAMP

Since many readers may not be familiar with PBiGAMP [21], we now briefly review the background of the algorithm in this subsection. PBiGAMP is a computational efficient approach to approximating the marginal posteriors of independent random variables ${\left\{x_{n}\right\}}_{n=0}^{N-1}$ and ${\left\{h_{l}\right\}}_{l=0}^{L-1}$ from measurements $y={\left[y_{0},\ldots ,y_{M-1}\right]}^{T}$ generated under a likelihood of the form

(8) $$\begin{array}{cc} p_{y\;|\;z}\left(y\;|\;z\right) & =\prod_{m=0}^{M-1}p_{y_{m}\;|\;z_{m}}\left(y_{m}\;|\;z_{m}\right) \end{array}$$

where the noiseless observation $z_{m}$ is

(9) $$\begin{array}{cc} z_{m} & =\sum_{n=0}^{N-1}\sum_{l=0}^{L-1}x_{n}z_{m}^{\left(n,l\right)}h_{l},, \end{array}$$

with known parameter $z_{m}^{\left(n,l\right)}$ determined by the system. PBiGAMP assumes that x and h obey independent distribution, for example,

(10) $$\begin{array}{cc} p_{x}\left(x\right) & =\prod_{n=0}^{N-1}p_{x_{n}}\left(x_{n}\right), \end{array}$$

(11) $$\begin{array}{cc} p_{h}\left(h\right) & =\prod_{L=0}^{L-1}p_{h_{l}}\left(h_{l}\right).. \end{array}$$

Note that to apply PBiGAMP, we should specify what $z_{m}^{\left(n,l\right)}$, $p_{y_{m}\;|\;z_{m}}\left(y_{m}\;|\;z_{m}\right)$, $p_{x_{n}}\left(x_{n}\right)$ and $p_{h_{l}}\left(h_{l}\right)$ are in SC system.

**Figure 3:**
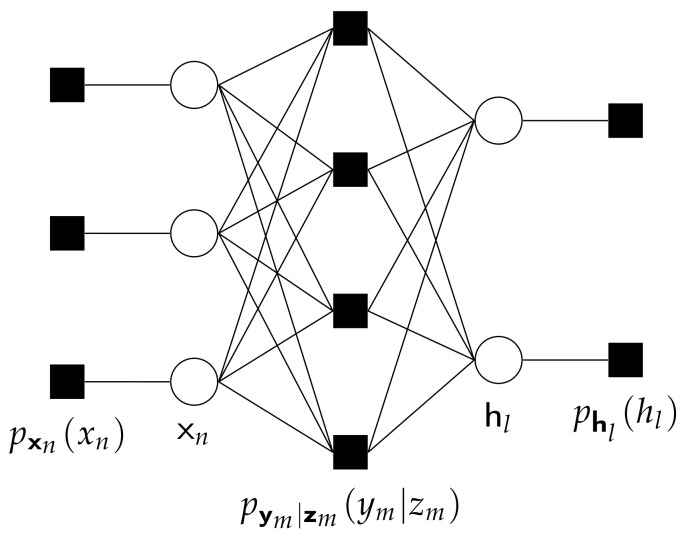
The factor graph for parametric generalized bilinear inference under N=3, M=4, and L=2.

The factor graph for PBiGAMP is shown in Figure 3. The main ideas behind PBiGAMP are the followings. First, although the messages flowing rightward from nodes $\left\{x_{n}\right\}$ to measurement nodes $\left\{p_{y_{m}\;|\;z_{m}}\left(y_{m}\;|\;z_{m}\right)\right\}$ and leftward from node $\left\{h_{l}\right\}$ to $\left\{p_{y_{m}\;|\;z_{m}}\left(y_{m}\;|\;z_{m}\right)\right\}$ are clearly non-Gaussian, PBiGAMP accurately approximates the messages about $z_{m}=\sum_{n=0}^{N-1}\sum_{l=0}^{L-1}x_{n}z_{m}^{\left(n,l\right)}h_{l}$ as Gaussian, when *N* and *L* are large, using the central limit theorem. Moreover, to obtain the parameters of the distribution about $z_{m}$ (i.e., its mean and variance), only the mean and variance of each $x_{n}$ and $h_{l}$ are needed. Thus, it suffices to pass only means and variances rightward from each $x_{n}$ and leftward from each $h_{l}$. Second, since the measurement nodes $\left\{p_{y_{m}\;|\;z_{m}}\left(y_{m}\;|\;z_{m}\right)\right\}$ are probably non-Gaussian (i.e., the quantized model described in Section 2.1), the messages from measurement nodes $\left\{p_{y_{m}\;|\;z_{m}}\left(y_{m}\;|\;z_{m}\right)\right\}$ flowing leftward to $\left\{x_{n}\right\}$ and rightward to $\left\{h_{l}\right\}$ would be non-Gaussian. PBiGAMP approximates them as Gaussian using the second-order Taylor series, and pass only the resulting means and variances leftward (rightward) from measurement nodes $\left\{p_{y_{m}\;|\;z_{m}}\left(y_{m}\;|\;z_{m}\right)\right\}$ to $\left\{x_{n}\right\}$ ($\left\{h_{l}\right\}$) nodes. Finally, PBiGMAP employs further simplifications to approximate differences among the outgoing means and variances of each measurement nodes $\left\{p_{y_{m}\;|\;z_{m}}\left(y_{m}\;|\;z_{m}\right)\right\}$, and the incoming means and variances of each variable nodes $\left\{x_{n}\right\}$ and $\left\{h_{l}\right\}$, using the first-order Taylor series approximation. Additionally, PBiGAMP repeatedly drops terms that vanish in the large-system limit.

### 3.2. Joint Symbol Detection, Channel Estimation and Decoding Scheme via PBiGAMP

To derive the proposed joint symbol-detection, channel-estimation and decoding framework, we should first specify the symbol prior $p_{x_{n}}\left(x_{n}\right)$ in Equation (10), the channel prior $p_{h_{l}}\left(h_{l}\right)$ in Equation (11), the likelihood function $p_{y_{m}\;|\;z_{m}}\left(y_{m}/z_{m}\right)$ in Equation (8) and the PBiGAMP quantity $z_{m}^{\left(i,j\right)}$ in Equation (9).

Due to the sparse behavior of mmWave channels, we propose to use *D*-state Gaussian mixture model (GMM) [8,20] to estimate channels,

(12) $$\begin{array}{c} p_{h_{l}}\left(h_{l}\right)=\sum_{d=1}^{D}\lambda_{l,d}\mathrm{CN}\left(h_{l};0,\nu_{l,d}\right), \end{array}$$

where $\lambda_{l,d}\geq 0$ and $\nu_{l,d}>0$ are the weight and variance of the *d*-th mixture component of the *l* tap, and we have $\sum_{d=1}^{D}\lambda_{l,d}=1\;\forall l$. Note that $p_{h_{l}}\left(h_{l}\right)$ can be treated as the yellow left arrow in Figure 2.

For PBiGAMP’s prior on $x_{n}$, we align

(13) $$\begin{array}{cc} p_{x_{n}}\left(x_{n}\right)\; & =\sum_{j=1}^{2^{A}}\gamma_{n,j}\delta \left(x_{n}-s^{\left(j\right)}\right), \end{array}$$

where δ(·) is the Dirac delta, $\{s^{\left(1\right)},\ldots ,s^{\left(2^{A}\right)}\}≜S$ is the data-symbol alphabet, and $\gamma_{n,j}=\mathrm{Pr}\left\{x_{n}\;=\;s^{\left(j\right)}\right\}$ is the prior data-symbol probability mass function (pmf), which is determined by the coded bit priors $\mathrm{Pr}\{c_{n,a}=c_{a}^{\left(j\right)}\}$ coming from the soft decoder, that is,

(14) $$\begin{array}{cc} \gamma_{n,j}\; & ≜\mathrm{Pr}\left\{x_{n}\;=\;s^{\left(j\right)}\right\}=\sum_{j^{\prime }=1}^{2^{A}}\mathrm{Pr}\left\{x_{n}\;=\;s^{\left(j\right)},c_{n}=c^{\left(j^{\prime }\right)}\right\} \end{array}$$

(15) $$\begin{array}{cc} \; & =\sum_{j^{\prime }=1}^{2^{A}}\underset{\delta_{j-j^{\prime }}}{\underset{︸}{\mathrm{Pr}\{x_{n}\;=\;s^{\left(j\right)}\;|\;c_{n}=c^{\left(j^{\prime }\right)}\}}}\mathrm{Pr}\left\{c_{n}=c^{\left(j^{\prime }\right)}\right\} \end{array}$$

(16) $$\begin{array}{cc} \; & =\mathrm{Pr}\left\{c_{n}=c^{\left(j\right)}\right\}=\prod_{a=1}^{A}\mathrm{Pr}\left\{c_{n,a}=c_{a}^{\left(j\right)}\right\}, \end{array}$$

where $c^{\left(j\right)}={\left[c_{1}^{\left(j\right)},\ldots ,c_{A}^{\left(j\right)}\right]}^{T}\in {\left\{0,1\right\}}^{A}$ is the coded-bit sequence corresponding to the symbol value $s^{\left(j\right)}$, and $\delta_{j}$ is the Kronecker delta sequence. Note that $p_{x_{n}}\left(x_{n}\right)$ can be treated as the green right arrow in Figure 2.

For likelihood function $p_{y_{m}\;|\;z_{m}}\left(y_{m}/z_{m}\right)$, we have

(17) $$\begin{array}{c} p_{y_{m}\;|\;z_{m}}\left(y_{m}\;|\;z_{m}\right)=\int_{Q^{-1}\left(y_{m}\right)}\mathrm{CN}(w;z_{m},\sigma_{w}^{2})\;dw, \end{array}$$

where $Q^{-1}\left(y_{m}\right)\subset C$ is the region quantized to $y_{m}$.

As for PBiGAMP quantity $z_{m}^{\left(n,l\right)}$, due to the fact that the circulant channel matrix can be decomposed as $H=\sum_{l=0}^{L-1}h_{l}Jl$ with the *l*-circulant delay matrix $Jl\in {\left\{0,1\right\}}^{M\times M}$, we can rewrite Equation (3) as

(18) $$\begin{array}{cc} y_{m} & =Q\left(\sum_{l=0}^{L-1}\sum_{n=0}^{MK-1}h_{l}z_{m}^{\left(n,l\right)}x_{n}+w_{m}\right), \end{array}$$

where we define

(19) $$\begin{array}{cc} z_{m}^{\left(n,l\right)} & ≜{\left[I_{K}⊗Jl\right]}_{m,n}. \end{array}$$

We are now ready to design the joint symbol-detection, channel-estimation and decoding framework based on PBiGAMP. Roughly speaking, messages are passed on the factor graph in Figure 2 from the left to the right and back again, several times, stopping once the messages converge. One such forward-backward pass will be referred as a “Turbo iteration”. Furthermore, during a single Turbo iteration, there are multiple internal iterations of message passing within soft PBiGAMP equalization sub-graphs, which will be referred to as “PBiGAMP iteration”. Finally, SISO decoding sub-graphs may itself be implemented using message passing with several internal iterations.

Next, we will describe the design of soft PBiGAMP equalization, especially how to deal with the non-linear procedures from quantized likelihood in Equation (17), discrete symbols’ prior in Equation (13) and GMM channels’ prior in Equation (12).

As described in Section 3.1, during each PBiGAMP iteration, PBiGMAP treats $z_{m}$ as Gaussian under large *L*, *K* and *M*, whose mean and variance are denoted by ${\hat{p}}_{m}$ and $\nu^{p}$, respectively, that is, $p_{z_{m}}\left(z_{m}\right)=\mathrm{CN}\left(z_{m};{\hat{p}}_{m},\nu^{p}\right)$. Along with the quantized likelihood defined in Equation (17), PBiGAMP can reach the approximation of the true marginal posterior pdf of $z_{m}$

(20) $$\begin{array}{cc} p_{z_{m}\;|\;y_{m}}\left(z_{m}\;|\;y_{m}\right) & =\frac{p_{z_{m}}\left(z_{m}\right)p_{y_{m}\;|\;z_{m}}\left(y_{m}\;|\;z_{m}\right)}{\int p_{z_{m}}\left(z_{m}\right)p_{y_{m}\;|\;z_{m}}\left(y_{m}\;|\;z_{m}\right)\;dz_{m}} \end{array}$$

(21) $$\begin{array}{cc} \; & =\frac{\mathrm{CN}\left(z_{m};{\hat{p}}_{m},\nu^{p}\right)\int_{Q^{-1}\left(y_{m}\right)}\mathrm{CN}(w;z_{m},\sigma_{w}^{2})\;dw}{\int \mathrm{CN}\left(z_{m};{\hat{p}}_{m},\nu^{p}\right)\int_{Q^{-1}\left(y_{m}\right)}\mathrm{CN}(w;z_{m},\sigma_{w}^{2})\;dw\;dz_{m}}. \end{array}$$

One can then obtain the minimum mean square error (MMSE) estimate and estimate variance (For the purpose of low-complexity, we consider the scalar-variance version of PBiGAMP.) of $z_{m}$ via

(22) $$\begin{array}{cc} {\hat{z}}_{m} & =E\left[z_{m}\;|\;{\hat{p}}_{m};\nu^{p}\right]=\int z_{m}p_{z_{m}\;|\;y_{m}}\left(z_{m}\;|\;y_{m}\right)\;dz_{m}, \end{array}$$

(23) $$\begin{array}{cc} \nu^{z} & =\frac{1}{MK}\sum_{m=0}^{MK-1}\underset{≜\nu_{m}^{z}}{\underset{︸}{\mathrm{var}[z_{m}\;|\;{\hat{p}}_{m};\nu^{p}]}}=\frac{1}{MK}\sum_{m=0}^{MK-1}\int {\left|z_{m}-{\hat{z}}_{m}\right|}^{2}p_{z_{m}\;|\;y_{m}}\left(z_{m}\;|\;y_{m}\right)\;dz_{m}. \end{array}$$

Note that the real and imaginary part of $z_{m}$ are independent Gaussian with mean p^mRe and p^mIm, respectively, and variance $\frac{\nu^{p}}{2}$. Since Q(·) quantizes the real and imaginary part separately as shown in Figure 1, we can also separately compute posterior mean and variance of the real and imaginary part of $z_{m}$. Denoting the interval of y¯mRe quantized to ymRe by $(g_{u-1},g_{u}]\subset R$, plugging Equation (21) into Equations (22) and (23) yields the posterior mean and variance of the real part of $z_{m}$

(24) z^mRe=p^mRe+νp2DmReEmRe,

(25) νmz,Re=νp2+FmReEmReνp22−(z^mRe−p^mRe)2,

where

(26) DmRe=Np^mRe−gu−1;0,(σw2+νp)/2−Np^mRe−gu;0,(σw2+νp)/2,

(27) EmRe=Φp^mRe−gu−1(σw2+νp)/2−Φp^mRe−gu−1(σw2+νp)/2,FmRe=p^mRe−gu(σw2+νp)/2Np^mRe−gu;0,(σw2+νp)/2

(28) −p^mRe−gu−1(σw2+νp)/2Np^mRe−gu−1;0(σw2+νp)/2.

We can obtain the posterior mean and variance of the imaginary part of $z_{m}$ in the similar way by replacing the superscript “Re” with “Im” in Equations (24)–(28). Finally, combining the real and imaginary part will lead to the posterior mean and variance of $z_{m}$

(29) z^m=z^mRe+jz^mIm,

(30) νz=1MK∑m=0MK−1νmz,Re+νmz,im.

See Reference [16] (Appendix A]) for the further details to derive Equations (24)–(28).

In the sequel, based on $\left\{{\hat{z}}_{m},\nu^{z}\right\}$, PBiGAMP will produce quantities $\left\{{\hat{r}}_{l},\nu^{r}\right\}$, such that ${\hat{r}}_{l}$ behaves like a white Gaussian noise corrupted version of the true channel tap $h_{l}$. That is,

(31) $$\begin{array}{c} {\hat{r}}_{l}=h_{l}+\sqrt{\nu^{r}}e, \end{array}$$

where *e* is a zero-mean Gaussian random variable with unit variance. Based on the above model Equation (31) and the GMM prior in Equation (12), PBiGAMP can approximate the true posterior pdf of $h_{l}$ as

(32) $$\begin{array}{cc} p_{h_{l}\;|\;r_{l}}\left(h_{l}\;|\;{\hat{r}}_{l}\right)\; & =\frac{p_{h_{l}}\left(h_{l}\right)p_{r_{l}\;|\;h_{l}}\left({\hat{r}}_{l}\;|\;h_{l}\right)}{\int p_{h_{l}}\left(h_{l}\right)p_{r_{l}\;|\;h_{l}}\left({\hat{r}}_{l}\;|\;h_{l}\right)\;dh_{l}} \end{array}$$

(33) $$\begin{array}{cc} \; & =\sum_{d=1}^{D}{\bar{\lambda }}_{l,d}\mathrm{CN}\left(h_{l};\frac{\nu_{l,d}{\hat{r}}_{l}}{\nu_{l,d}+\nu^{r}},\frac{\nu_{l,d}\nu^{r}}{\nu_{l,d}+\nu^{r}}\right), \end{array}$$

where

(34) $$\begin{array}{cc} {\bar{\lambda }}_{l,d}\; & =\frac{\lambda_{l,d}\mathrm{CN}\left({\hat{r}}_{l};0,\nu_{l,d}+\nu^{r}\right)}{\sum_{d^{\prime }=1}^{D}\lambda_{l,d^{\prime }}\mathrm{CN}\left({\hat{r}}_{l};0,\nu_{l,d^{\prime }}+\nu^{r}\right)}. \end{array}$$

In Equation (32), $p_{r_{l}\;|\;h_{l}}\left({\hat{r}}_{l}\;|\;h_{l}\right)=\mathrm{CN}\left(h_{l};{\hat{r}}_{l},\nu^{r}\right)$ can be seen from Equation (31), acting as the likelihood pdf. Note that $p_{r_{l}\;|\;h_{l}}\left({\hat{r}}_{l}\;|\;h_{l}\right)=\mathrm{CN}\left(h_{l};{\hat{r}}_{l},\nu^{r}\right)$ can be interpreted as the product of blue right arrows in Figure 2. One then can obtain the MMSE estimate and estimate variance of $h_{l}$ via

(35) $$\begin{array}{cc} {\hat{h}}_{l} & =E\left[h_{l}\;|\;{\hat{r}}_{l};\nu^{r}\right]=\int h_{l}p_{h_{l}\;|\;r_{l}}\left(h_{l}\;|\;{\hat{r}}_{l}\right)\;dh_{l} \end{array}$$

(36) $$\begin{array}{cc} \; & =\sum_{d=1}^{D}{\bar{\lambda }}_{l,d}\frac{\nu_{l,d}{\hat{r}}_{l}}{\nu_{l,d}+\nu^{r}}, \end{array}$$

(37) $$\begin{array}{cc} \nu_{l}^{h} & =\frac{1}{L}\sum_{l=0}^{L-1}\mathrm{var}\left[h_{l}\;|\;{\hat{r}}_{l};\nu^{r}\right]=\frac{1}{L}\sum_{l=0}^{L-1}\int {\left|h_{l}-{\hat{h}}_{l}\right|}^{2}p_{h_{l}\;|\;r_{l}}\left(h_{l}\;|\;{\hat{r}}_{l}\right)\;dh_{l} \end{array}$$

(38) $$\begin{array}{cc} \; & =\frac{1}{L}\sum_{l=0}^{L-1}\left[\sum_{d=1}^{D}{\bar{\lambda }}_{l,d}\left(\frac{\nu_{l,d}\nu^{r}}{\nu_{l,d}+\nu^{r}}+|\frac{\nu_{l,d}{\hat{r}}_{l}}{\nu_{l,d}+\nu^{r}}{|}^{2}\right)-{\left|{\hat{h}}_{l}\right|}^{2}\right]. \end{array}$$

Similarly, PBiGAMP also produces quantities $\left\{{\hat{q}}_{n},\nu^{q}\right\}$, such that ${\hat{q}}_{n}$ behaves like a white Gaussian noise corrupted version of the true symbol $x_{n}$. That is

(39) $$\begin{array}{c} {\hat{q}}_{n}=x_{n}+\sqrt{\nu^{q}}e. \end{array}$$

Based on above model and discrete prior on symbols in Equation (13), PBiGAMP then approximate the true posterior pdf of $x_{n}$ as

(40) $$\begin{array}{cc} p_{x_{n}\;|\;q_{n}}\left(x_{n}\;|\;{\hat{q}}_{n}\right)\; & =\frac{p_{x_{n}}\left(x_{n}\right)p_{q_{n}\;|\;x_{n}}\left({\hat{q}}_{n}\;|\;x_{n}\right)}{\int p_{x_{n}}\left(x_{n}\right)p_{q_{n}\;|\;x_{n}}\left({\hat{q}}_{n}\;|\;x_{n}\right)\;dx_{n}} \end{array}$$

(41) $$\begin{array}{cc} \; & =\sum_{j=1}^{2^{A}}{\bar{\gamma }}_{n,j}\delta \left(x_{n}-s^{\left(j\right)}\right), \end{array}$$

where

(42) $$\begin{array}{cc} {\bar{\gamma }}_{n,j}\; & =\frac{\mathrm{Pr}\left\{x_{n}\;=\;s^{\left(j\right)}\right\}\mathrm{CN}\left(s^{\left(j\right)};{\hat{q}}_{n},\nu^{q}\right)}{\sum_{j^{\prime }=1}^{2^{A}}\mathrm{Pr}\left\{x_{n}\;=\;s^{\left(j^{\prime }\right)}\right\}\mathrm{CN}\left(s^{\left(j^{\prime }\right)};{\hat{q}}_{n},\nu^{q}\right)}. \end{array}$$

In Equation (40), $p_{q_{n}\;|\;x_{n}}\left({\hat{q}}_{n}\;|\;x_{n}\right)=\mathrm{CN}\left(x_{n};{\hat{q}}_{n},\nu^{q}\right)$ can be seen from Equation (39), acting as the likelihood pdf. Note that $p_{q_{n}\;|\;x_{n}}\left({\hat{q}}_{n}\;|\;x_{n}\right)=\mathrm{CN}\left(x_{n};{\hat{q}}_{n},\nu^{q}\right)$ can be interpreted as the product of red left arrows in Figure 2. One then can obtain the MMSE estimate and estimate variance of $x_{n}$ via

(43) $$\begin{array}{cc} {\hat{x}}_{n} & =E\left[x_{n}\;|\;{\hat{q}}_{n};\nu^{q}\right]=\int x_{n}p_{x_{n}\;|\;q_{n}}\left(x_{n}\;|\;{\hat{q}}_{n}\right)\;dx_{n} \end{array}$$

(44) $$\begin{array}{cc} \; & =\sum_{j=1}^{2^{A}}{\bar{\gamma }}_{n,j}s^{\left(j\right)}, \end{array}$$

(45) $$\begin{array}{cc} \nu^{x} & =\frac{1}{MK}\sum_{n=0}^{MK-1}\mathrm{var}\left[x_{n}\;|\;{\hat{q}}_{n};\nu^{q}\right]=\frac{1}{MK}\sum_{n=0}^{MK-1}\int {\left|x_{n}-{\hat{x}}_{n}\right|}^{2}p_{x_{n}\;|\;q_{n}}\left(x_{n}\;|\;{\hat{q}}_{n}\right)\;dx_{n} \end{array}$$

(46) $$\begin{array}{cc} \; & =\frac{1}{MK}\sum_{n=0}^{MK-1}\sum_{j=1}^{2^{A}}{\bar{\gamma }}_{n,j}{\left|s^{\left(j\right)}-{\hat{x}}_{n}\right|}^{2}. \end{array}$$

Based on the above newly-computed quantities $\left\{{\hat{h}}_{l},\nu^{h}\right\}$ and $\left\{{\hat{x}}_{n},\nu^{x}\right\}$, PBiGAMP then updates $\left\{{\hat{p}}_{m},\nu^{p}\right\}$ and starts the next PBiGAMP iteration.

After the messages within the PBiGAMP equalization sub-graph have converged, PBiGAMP outputs quantities

(47) $$\begin{array}{cc} {\tilde{q}}_{n} & =\alpha {\hat{q}}_{n}+\left(1-\alpha \right){\hat{x}}_{n}, \end{array}$$

(48) $$\begin{array}{cc} {\tilde{\nu }}^{q} & =\alpha \nu^{q}+\left(1-\alpha \right)\nu^{x}, \end{array}$$

where $\left\{{\hat{q}}_{n},\nu^{q}\right\}$ and $\left\{{\hat{x}}_{n},\nu^{x}\right\}$ are collected from the latest PBiGAMP iteration, and α is the doping factor to control the weights of the “extrinsic” component $\left\{{\hat{q}}_{n},\nu^{q}\right\}$ and “posterior” component $\left\{{\hat{x}}_{n},\nu^{x}\right\}$. Note that our proposed scheme will reduce to the joint approach proposed in Reference [20] when α=1, and soft decoder will accept entire posterior information from soft PBiGAMP equalizer with α=0.

We then convert $\left\{{\tilde{q}}_{n},{\tilde{\nu }}^{q}\right\}$ into soft probabilities on coded bits via

(49) $$\begin{array}{cc} \mathrm{Pr}\{c_{n,a}\;=\;1\;|\;{\tilde{q}}_{n},\nu^{q}\}\; & =\sum_{j=1\ldots 2^{A}|c_{a}^{\left(j\right)}=1}\mathrm{Pr}\left\{c_{n}\;=\;c^{\left(j\right)}\;|\;{\tilde{q}}_{n},{\tilde{\nu }}^{q}\right\} \end{array}$$

(50) $$\begin{array}{cc} \; & =\sum_{j=1\ldots 2^{A}|c_{a}^{\left(j\right)}=1}{\bar{\gamma }}_{n,j}, \end{array}$$

where $c_{n}≜{\left[c_{n,1},\ldots ,c_{n,A}\right]}^{T}$ determines the value of data symbol $x_{n}$, and $c^{\left(j\right)}={\left[c_{1}^{\left(j\right)},\ldots ,c_{A}^{\left(j\right)}\right]}^{T}\in {\left\{0,1\right\}}^{A}$ is the coded-bit sequence corresponding to the symbol value $s^{\left(j\right)}$. The coded bit posteriors in Equation (50) are then converted to extrinsic form and passed to the SISO decoder. Finally, SISO decoder accepts this extrinsic information, treating it as a prior on the coded bits. It then outputs the posteriors on the coded bits, and converts them to extrinsic form, and updates $\gamma_{n,j}=\mathrm{Pr}\left\{x_{n}\;=\;s^{\left(j\right)}\right\}$ in Equation (16) for the next Turbo iteration. Since SISO decoding is a well-studied topic [25] and high-performance implementations are readily available [26], we will not elaborate on the details here.

The PBiGAMP-based soft equalizer procedure is summarized in Box 1, where we use (M×K)-matricized versions of $\hat{p}$, $\hat{q}$ and $\hat{x}$, denoted by $\hat{P}$, $\hat{Q}$ and $\hat{X}$, respectively. Note that we ignore explaining the linear steps in Box 1. Please see Reference [20] for further details. In the table, ⊙ means element-wise product, and index “t” means the PBiGAMP iteration number. We also summary the proposed joint symbol-detection, channel-estimation and decoding scheme in Box 2, where the index $“\bar{t}”$ means Turbo iteration number.

Box 1 **Soft PBiGAMP Equalizer.**

Definitions:

$$\begin{array}{c} \;p_{z_{m}|y_{m}\;}\left(z\;|\;\hat{p};\nu^{p}\right)\;≜\frac{p_{y_{m}|z_{m}\;}\left(y_{m}\;|\;z\right)\;\mathrm{CN}\left(z;\hat{p},\nu^{p}\right)}{\int p_{y_{m}|z_{m}\;}\left(y_{m}\;|\;z^{\prime }\right)\;\mathrm{CN}\left(z^{\prime };\hat{p},\nu^{p}\right)\;dz^{\prime }}\;(D1)\\ p_{h_{l}|r_{l}\;}\left(h\;|\;\hat{r};\nu^{r}\right)\;≜\frac{p_{h_{l}\;}\left(h\right)\;\mathrm{CN}\left(\hat{r};h,\nu^{r}\right)}{\int p_{h_{l}\;}\left(h^{\prime }\right)\;\mathrm{CN}\left(\hat{r};h^{\prime },\nu^{r}\right)\;dh^{\prime }}\;(D2)\\ p_{x_{n}|q_{n}\;}\left(x\;|\;\hat{q};\nu^{q}\right)\;≜\frac{p_{x_{n}\;}\left(x\right)\;\mathrm{CN}\left(\hat{q};x,\nu^{q}\right)}{\int p_{x_{n}\;}\left(x^{\prime }\right)\;\mathrm{CN}\left(\hat{q};x^{\prime },\nu^{q}\right)\;dx^{\prime }}\;(D3) \end{array}$$

Initialization:

$$\begin{array}{c} \hat{X}\left[1\right]=O_{M\times K},\;\nu^{x}\left[1\right]=1\\ \hat{h}\left[1\right]={\hat{h}}_{\mathrm{init}},\;\nu^{h}\left[1\right]=\nu_{\mathrm{init}}^{h},\;\hat{S}\left[0\right]=0_{M\times K} \end{array}$$

For $t=1,\ldots T_{\max}$

$$\begin{array}{cccc} \underline{\hat{X}}\left[t\right] & = & F_{M}\hat{X}\left[t\right] & \left(L1\right)\\ \underline{\hat{h}}\left[t\right] & = & F_{M}^{1:L}\hat{h}\left[t\right] & \left(L2\right)\\ {\bar{\nu }}^{p}\left[t\right] & = & \nu^{x}\left[t\right]∥\hat{h}\left[t\right]{∥}^{2}+\frac{L}{MK}\nu^{h}\left[t\right]∥\hat{X}\left[t\right]{∥}_{F}^{2} & \left(L3\right)\\ \nu^{p}\left[t\right] & = & {\bar{\nu }}^{p}\left[t\right]+L\nu^{h}\left[t\right]\nu^{x}\left[t\right] & \left(L4\right)\\ \hat{P}\left[t\right] & = & \sqrt{M}F_{M}^{H}\mathrm{Diag}\left(\underline{\hat{h}}\left[t\right]\right)\underline{\hat{X}}\left[t\right]-{\bar{\nu }}^{p}\left[t\right]\hat{S}\left[t\;-\;1\right] & \left(L5\right)\\ \nu^{z}\left[t\right] & = & \frac{1}{MK}\sum_{m=1}^{M-1}\sum_{k=1}^{K}\mathrm{var}\left\{z_{mk}\;|\;{\hat{p}}_{mk}\left[t\right];\nu^{p}\left[t\right]\right\} & \left(L6\right)\\ \forall m,k\;:{\hat{z}}_{mk}\left[t\right] & = & E[z_{mk}\;|\;p_{mk}\;=\;{\hat{p}}_{mk}\left[t\right];\nu^{p}\left[t\right]] & \left(L7\right)\\ \nu^{s}\left[t\right] & = & \left(1-\nu^{z}\left[t\right]/\nu^{p}\left[t\right]\right)/\nu^{p}\left[t\right] & \left(L8\right)\\ \hat{S}\left[t\right] & = & \left(\hat{Z}\left[t\right]-\hat{P}\left[t\right]\right)/\nu^{p}\left[t\right] & \left(L9\right)\\ \underline{\hat{S}}\left[t\right] & = & F_{M}\hat{S}\left[t\right] & \left(L10\right)\\ \nu^{r}\left[t\right] & = & {\left(\nu^{s}\left[t\right]∥\hat{X}\left[t\right]{∥}_{F}^{2}\right)}^{-1} & \left(L11\right)\\ \hat{r}\left[t\right] & = & \nu^{r}\left[t\right]\sqrt{M}{\left(F_{M}^{1:L}\right)}^{H}\left(\underline{\hat{X}}{\left[t\right]}^{*}⊙\underline{\hat{S}}\left[t\right]\right)1_{K}\\ \; & \; & +\left(1-MK\nu^{r}\left[t\right]\nu^{x}\left[t\right]\nu^{s}\left[t\right]\right)\hat{h}\left[t\right] & \left(L12\right)\\ \nu^{q}\left[t\right] & = & {\left(\nu^{s}\left[t\right]∥\hat{h}\left[t\right]{∥}^{2}\right)}^{-1} & \left(L13\right)\\ \hat{Q}\left[t\right] & = & \sqrt{M}\nu^{q}\left[t\right]F_{M}^{H}\mathrm{Diag}{\left(\underline{\hat{h}}\left[t\right]\right)}^{H}\underline{\hat{S}}\left[t\right]+\left(1-L\nu^{q}\left[t\right]\nu^{h}\left[t\right]\nu^{s}\left[t\right]\right)\hat{X}\left[t\right] & \left(L14\right)\\ \nu^{h}\left[t\;+\;1\right] & = & \frac{1}{L}\sum_{l=0}^{L-1}\mathrm{var}\left\{h_{l}\;|\;r_{l}={\hat{r}}_{l}\left[t\right];\nu^{r}\left[t\right]\right\} & \left(L15\right)\\ \forall l\;:{\hat{h}}_{l}\left[t\;+\;1\right] & = & E[h_{l}\;|\;r_{l}\;=\;{\hat{r}}_{l}\left[t\right];\nu^{r}\left[t\right]] & \left(L16\right)\\ \nu^{x}\left[t\;+\;1\right] & = & \frac{1}{MK}\sum_{m=0}^{M-1}\sum_{k=1}^{K}\mathrm{var}\left\{x_{mk}\;|\;{\hat{q}}_{mk}\left[t\right];\nu^{q}\left[t\right]\right\} & \left(L17\right)\\ \forall m,k\;:{\hat{x}}_{mk}\left[t\;+\;1\right] & = & E[x_{mk}\;|\;q_{mk}\;=\;{\hat{q}}_{mk}\left[t\right];\nu^{q}\left[t\right]] & \left(L18\right) \end{array}$$

end

Box 2 **The Proposed Joint Symbol-Detection, Channel-Estimation and Decoding Scheme.**

For $\bar{t}=1,\ldots T_{\max}$

1 $\mathrm{SISO}\;\mathrm{decoder}\;\mathrm{accepts}\;\mathrm{the}\;\mathrm{extrinsic}\;\mathrm{version}\;\mathrm{of}\mathrm{Pr}\left\{c_{n,a}\;=\;1\;|\;{\tilde{q}}_{n},\nu^{q}\right\},\mathrm{and}\;\mathrm{outputs}\mathrm{Pr}\left\{c_{n,a}=c_{a}^{\left(j\right)}\right\}$.
2 $\mathrm{PBiGAMP}\;\mathrm{equalizer}\;\mathrm{accepts}\mathrm{Pr}\left\{c_{n,a}=c_{a}^{\left(j\right)}\right\},\mathrm{and}\;\mathrm{outputs}\left\{{\hat{q}}_{n},\nu^{q}\right\},\left\{{\hat{x}}_{n},\nu^{x}\right\}\mathrm{and}\left\{{\hat{h}}_{l},\nu^{h}\right\}.$
3 $\mathrm{Obtain}\{{\tilde{q}}_{n},{\tilde{\nu }}^{q}\}$ via *Equations* (47) *and* (48).
4 $\mathrm{Obtain}\mathrm{Pr}\{c_{n,a}\;=\;1\;|\;{\tilde{q}}_{n},\nu^{q}\}\mathrm{via}$*Equation* (50).

## 4. Simulation Results

Before showing the performance evaluation, we now briefly describe the benchmark methods used later. An alternative approach is to linearize the quantization model Equation (3) based on Bussgang’s theorem [27] by introducing additional quantization noise. In this way, Equation (3) can be approximated as

(51) $$\begin{array}{cc} y\; & =\left(1-\eta \right)\left(I_{K}⊗H\right)x+\tilde{w}, \end{array}$$

where η is the normalized mean square error defined as $\eta ≜E[|Q\left(\bar{y}\right)-\bar{y}{|}^{2}]/E[|\bar{y}{|}^{2}]$, which is fixed under certain quantization resolution. Bussgang’s theorem treats the equivalent noise $\tilde{w}$ as AWGN with variance $\sigma_{\tilde{w}}^{2}=\left(1-\eta \right)(\eta \sigma_{x}^{2}{E\{∥h∥}^{2}\}+\sigma_{w}^{2})$, where $\sigma_{x}^{2}$ is symbols’ average transmit power.

Based on above linear model Equation (51), we have two benchmark methods. One is to perform PBiGAMP directly in this linear model (denotes as “PBiGAMP-Bus”). Compared to the proposed scheme, changes manifest only in lines (L6)–(L7) of Box 1. The other is to perform pilot-aided channel estimation firstly. Treating the above channel estimate as the true channel, we then apply the well-known linear MMSE (LMMSE) equalizer (denoted as “LMMSE-Bus”) (Since the standard LMMSE equalizer requires matrix inverse, which incurs a complexity of $O(KM^{3})$ per block of KM symbols, we adopt the unit-variance approximated version of standard LMMSE equalizer [20], whose per-symbol complexity is O(logM) using FFT..) We denote our proposed algorithm as “PBiGAMP α=XX” in the later simulation, and show the performance of the proposed scheme with PCSI (denoted as “PCSI”) as a reference.

We now describe the simulation setup. Recalling the single-carrier block transmission model from Section 2.1, $N_{b}=3584$ information bits were coded at rate R=1/2 by an irregular low-density parity-check (LDPC) code with average column weight 3. The resulting $N_{c}=7168$ coded bits were then Gray-mapped to 1792 16-QAM symbols (i.e., A=4). For the channels, we adopted the 60 GHz WLAN model [28], where we used the “conference room” scenario at baud rate 1.76 GHz with default parameter settings. For the quantization precision, we choose b=3.

**Figure 4:**
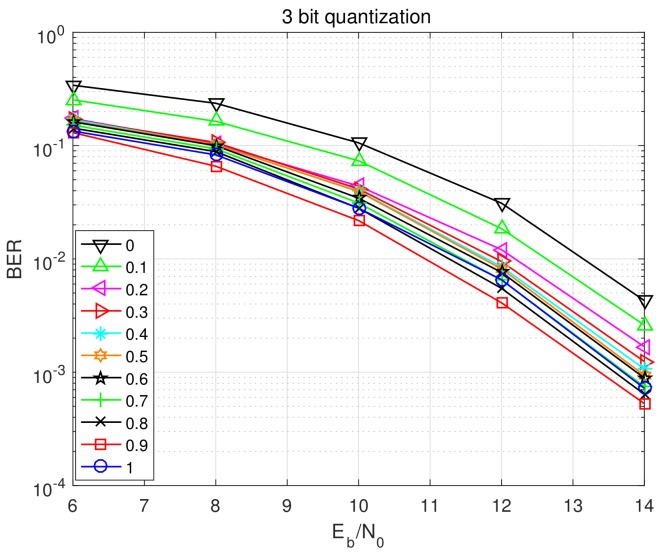
BER performance versus $E_{b}/N_{o}$ at 10-th Turbo iteration for different values of α.

**Figure 5:**
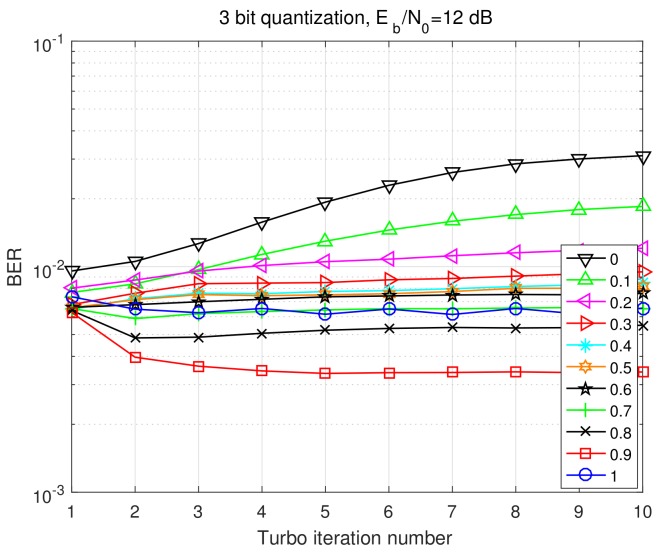
BER performance versus Turbo iteration number at $E_{b}/N_{o}=12$ dB for different values of α.

We first evaluate the bit error rate (BER) performances versus $E_{b}/N_{o}$ at 10-th Turbo iteration for different values of doping factor α as depicted in Figure 4. Interestingly, ′′α=0.9′′ trace shows the best performance, and its BER achieves about 2.1 dB better than that of the worst case ′′α=0′′, which implies the great influence of α on receiver performance. Compared to the PBiGAMP receiver [20] (the ′′α=1′′ trace), ′′α=0.9′′ can also beat it by about 0.4 dB performance gain. We further show the BER performance versus Turbo iteration number at $E_{b}/N_{o}=12$ dB for different values of doping factor α in Figure 5. Here we see that ′′α=0.9′′ significantly outperforms other traces. We also see that the BERs will get even worse with the increasing of Turbo iteration when choosing small α that is, 0–0.6. It is well known that Turbo principle implies to pass extrinsic information to SISO decoder. Here we introduce the doping factor to mix extrinsic information and posterior information, yielding better performance. In the mmWave systems with few-bit ADCs, there are deviations between the quantized receiving signals and unquantized receiving signals. Under this circumstance, introducing additional noise with certain level can sometimes improve the performance, which is referred as “stochastic resonance” phenomenon [29,30]. Here in our quantized SC system, we can regard the doping of posterior information as additional noise to dither the “pure extrinsic” information. In another words, the doping of posterior information can compensate the deviation from few-bit quantization. We use the doping factor α to control the level of this additional noise. Through Figure 4 and Figure 5, it can be seen that small level of additional noise (α=0.9,0.8) can help improve the performance.

**Figure 6:**
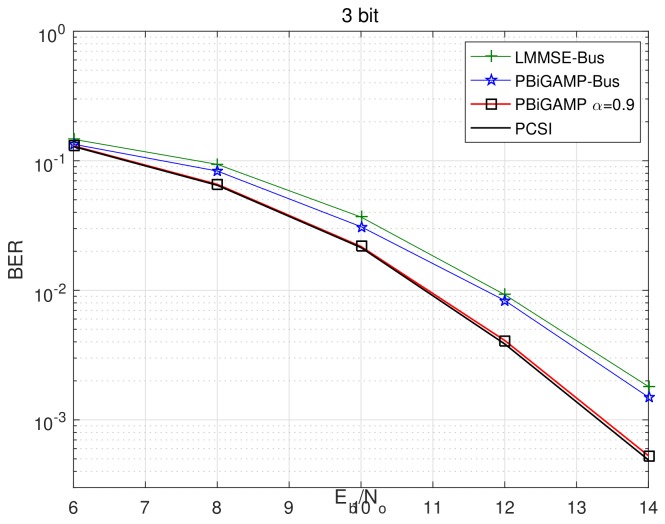
BER performance versus $E_{b}/N_{o}$ at 10-th Turbo iteration for investigated algorithms.

**Figure 7:**
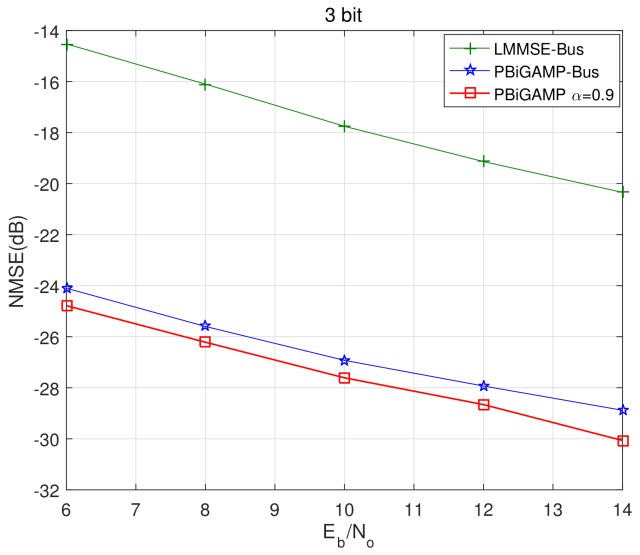
Channel estimation NMSE versus $E_{b}/N_{o}$ at 10-th Turbo iteration for investigated algorithms.

We then pick up the best trace ′′α=0.9′′, and compare it with PBiGAMP-Bus and LMMSE-Bus in Figure 6 and Figure 7. The BER performance is shown in Figure 6, where we can see that the BER of PBiGAMP α=0.9 is nearly indistinguishable from the PCSI bound, and outperforms about 1.1 dB and 1.3 dB better than that of PBiGAMP-Bus and LMMSE-Bus, respectively. The normalized mean square error (NMSE) of channel estimation is shown in Figure 7, where the NMSE of PBiGMAMP α=0.9 achieves about 2 dB and 10 dB better than that of PBiGAMP-Bus and LMMSE-Bus, respectively.

**Figure 8:**
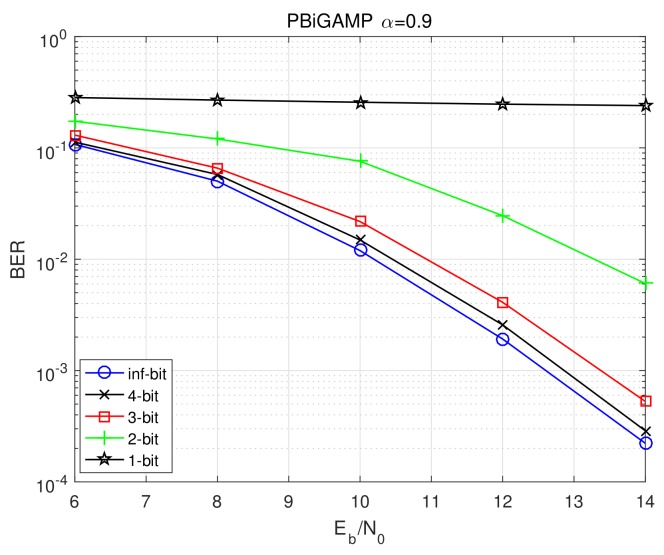
BER performance versus $E_{b}/N_{o}$ at 10-th Turbo iteration of the proposed algorithm under different quantization precisions.

For the proposed PBiGAMP receiver, we further show the BER performances versus $E_{b}/N_{o}$ at 10-th Turbo iteration under different quantization precisions in Figure 8 where “inf-bit” denotes no quantization. As we can see, the BERs get worse with the decrease of quantization precision. Compared to inf-bit case, the BERs of 4-bit and 3-bit degrade only about 0.2 dB and 0.8 dB, respectively; the BER of 2-bit case gets about 3.3 dB worse; and the BER of 1-bit case does not work well, which suggests to adopt stronger encoding or lower-order modulation (i.e., BPSK).

The complexity of the proposed PBiGAMP-based joint scheme is dominated by the DFT matrix multiplier in (L1), (L2), (L5), (L10), (L12) and (L14) in Box 1, which takes a total of Q(MKlogM) operations per iteration, or Q(logM) operations per symbol per iteration, via FFT. Due to the similarity between the proposed scheme and PBiGAMP-Bus, the complexity of PBiGAMP-Bus is also Q(logM) operations per symbol per iteration. As for LMMSE-Bus, since we adopt the unit-variance approximated version of LMMSE equalizer, whose complexity could reduce to Q(MKlogM) operations per iteration. Overall, the above three algorithms share the same level of complexity. The details about the number of FFT and the complexity of per-iteration for the four algorithms are shown in Table 1. Note that since the equalization part of LMMSE-Bus is not a self-iterative algorithm, we can not compute its per-iteration complexity.

## 5. Conclusions

In this paper, we considered mmWave single-carrier system under few-bit ADCs quantization, and proposed a joint symbol detection, channel estimation and decoding scheme based on PBiGAMP algorithm. Different form the common sense about Turbo equalization, our main contribution relies on the introduction of doping factor to combine “extrinsic” information and “posterior” information, which can include the joint approach in Reference [20] as a special case. Simulation results show that the significant performance gain can be achieved by our proposed scheme. The positive effect of doping comes from stochastic resonance, where the doping of posterior is regarded as additional noise to improve performance. Better understanding about the doping factor requires further investigation and we will study this point in our future work.

## Figures and Tables

**Table 1:** Complexity Comparison Between The Investigated Receivers.

	α=0.9	PBiGAMP in Reference [20]	PBiGAMP-Bus	LMMSE-Bus
Number of FFT	4K + 2	4K + 2	4K + 2	4K+1
Per-iteration Complexity	Q(MKlogM)	Q(MKlogM)	Q(MKlogM)	

## Abbreviations

The following abbreviations are used in this manuscript:

LTE	Long Term Evolution
ADC	analog-to-digital
PBiGAMP	Parameteric bilinear generalized approximate message passing
OFDM	orthogonal frequency division multiplexing
SC	single-carrier
FDE	frequency domain equalization
GAMP	generalized approximate message passing
VAMP	vector approximate message passing
MIMO	multiple input multiple output
PCSI	perfect channel state information
FFT	fast Fourier transform
pdf	probability density function
pmf	probability mass function
VGA	variable gain amplifier
AGC	automatic gain control
RF	radio frequency
BP	belief propagation
SPA	sum-product algorithm
MSE	mean square error
AWGN	additive white Gaussian noise
GMM	Gaussian mixture model
SISO	soft-input and soft-output
LDPC	low-density parity-check
MMSE	minimum mean square error
LMMSE	linear minimum mean square error
BER	bit error rate
NMSE	normalized mean square error
